# Isolated Superior Mesenteric Artery Dissection: A Novel Etiology and a Review

**DOI:** 10.3400/avd.ra.21-00055

**Published:** 2022-03-25

**Authors:** Rakan Nasser Eldine, Hassan Dehaini, Jamal Hoballah, Fady Haddad

**Affiliations:** 1 Division of Vascular & Endovascular Surgery, Department of Surgery, Faculty of Medicine, American University of Beirut, Beirut, Lebanon

**Keywords:** ISMAD, blunt trauma, endovascular, dissection, superior mesenteric artery

## Abstract

Isolated superior mesenteric artery dissection (ISMAD) is a rare pathology with multifactorial etiology. The aim of this article is to provide a narrative review of the latest literature about ISMAD. Case reports, series, and recent meta-analyses were included. This review is introduced with a brief case report of a rare etiology of ISMAD, followed by a discussion of its etiology, clinical presentation, diagnosis, classification, and treatment, and we report a new cause of ISMAD, that is, blunt abdominal trauma. The etiology of ISMAD is multifactorial, consisting of anatomic, genetic, and systemic components. ISMAD is more common among middle-aged males and in East Asia. Its clinical presentation ranges from asymptomatic to mesenteric ischemia, albeit mortality remains <1%. It is diagnosed and classified mostly by computed tomography angiography, and there are five classification systems for ISMAD, though traumatic etiology may be added. The treatment of ISMAD is mostly conservative, with a success rate exceeding 90%. Endovascular stenting is second line, reserved so far for failed medical management, though its role is expanding to include earlier management of symptomatic patients, while open surgical repair is left for acute mesenteric ischemia with bowel compromise.

## Introduction

**Figure 1:**
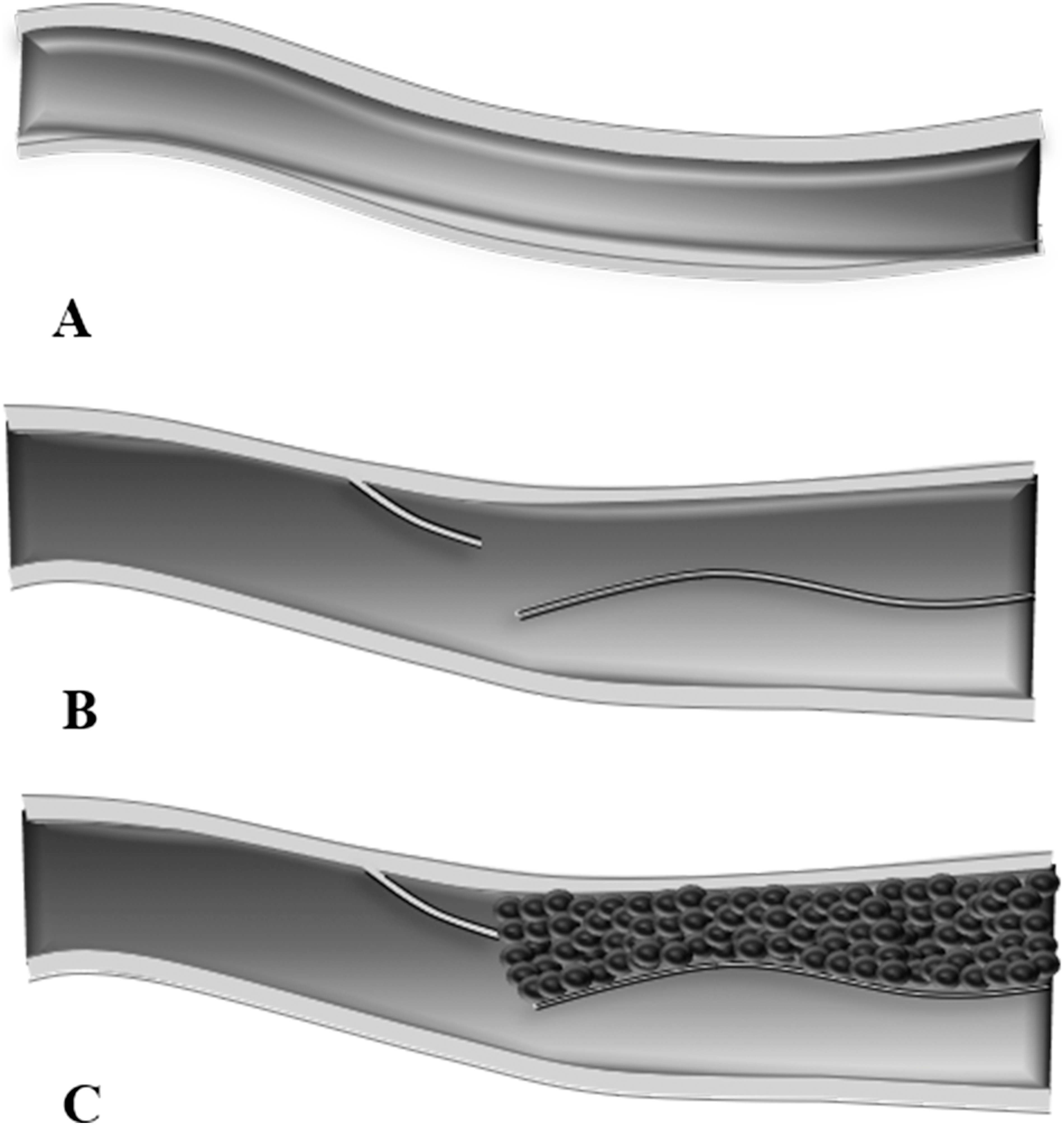
Fig. 1 (**A**) A patent artery. (**B**) Arterial dissection with patent true and false lumen. (**C**) Arterial dissection with patent true lumen and thrombosed false lumen.

Arterial dissection is a disruption in the arterial wall layers that starts with an intimal tear and progresses to bleeding within the tunica media, forming a false lumen adjacent to the true one. The dissecting flap can obscure orifices of branching arteries, resulting in ischemia of distal organs, while the blood within the false lumen can coagulate and precipitate thromboembolism (**Fig. 1**). Moreover, arterial dissection can damage the tunica adventitia and lead to arterial rupture.^1)^ Although arterial dissection is commonly associated with the aorta, any artery is at risk, such as the superior mesenteric artery (SMA).

SMA dissection is usually an extension of aortic dissection,^2)^ yet isolated spontaneous SMA dissection (ISMAD) has been reported since 1947,^3)^ either as a symptomatic entity or an incidental finding. It remains the most common of the mesenteric vessel dissections.^4,5)^ As cases of spontaneous ISMAD continue to be discovered in an increasing manner due to advancement in radiographic technology, this article reviews the most recent updates about ISMAD while simultaneously reporting a novel etiology where ISMAD is secondary to blunt abdominal trauma (BAT).

## Case Presentation

A 59-year-old male, smoker, known to have Hashimoto thyroiditis and dyslipidemia, presented to our clinic for abdominal pain that lasted for three weeks. The pain was diffuse, non-radiating, intermittent, and moderately severe. The patient’s vitals were normal, and his physical examination was non-revealing.

Probing more into his history, the patient recalled sustaining a BAT at work, localized to the epigastric region; he tripped and fell onto the corner of a large solid equipment. Initially, the pain was severe, but its intensity abated with time. Nevertheless, the persistence of his pain despite analgesics for 3 days drove him to seek medical advice at another institution, where a computed tomography (CT) of his abdomen, esophagogastroduodenoscopy, and colonoscopy were done. The patient was diagnosed with uncomplicated, acute partial superior mesenteric vein thrombosis and was started on rivaroxaban 15 mg twice daily, which failed to improve his symptoms after three weeks.

**Figure 2:**
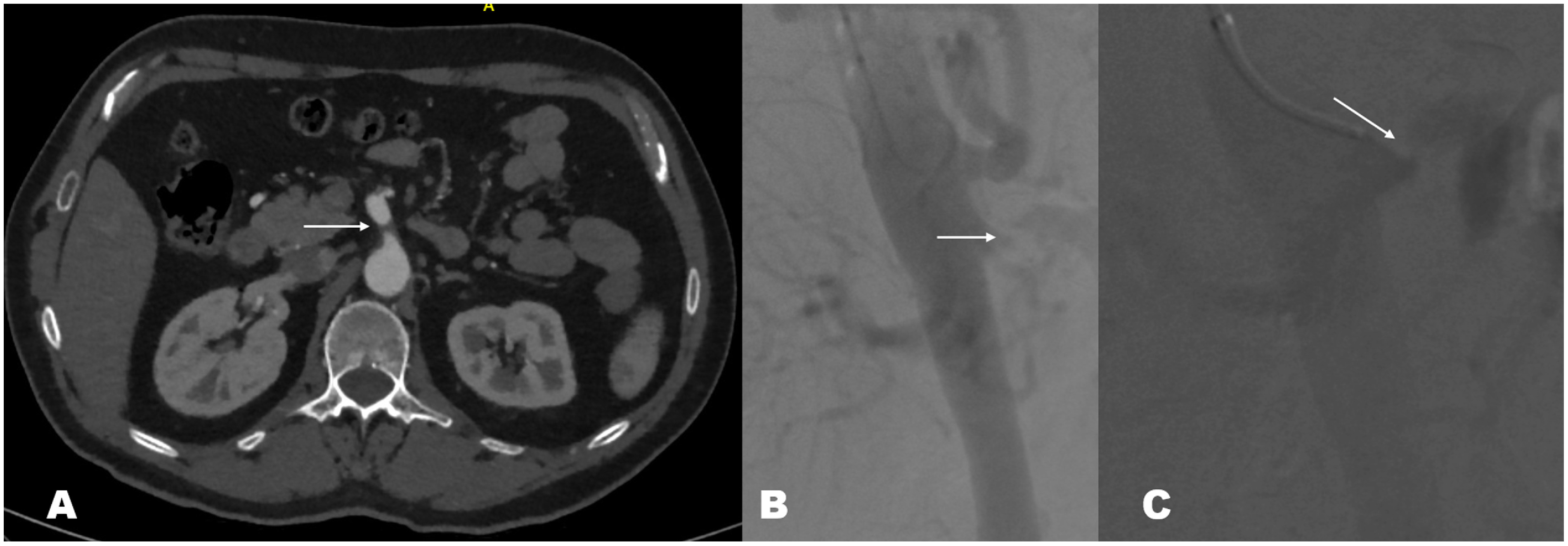
Fig. 2 (**A**) Computed tomography angiography showing a stenosed superior mesenteric artery with distal perfusion. There is no embolus nor atherosclerotic changes surrounding the lesion. (**B**) Nonselective angiography showing isolated superior mesenteric artery dissection (ISMAD). (**C**) Selective angiography showing ISMAD.

At our clinic, review of the CT scans was not convincing because only 5-mm cuts were provided. A new thin-slice triple phase CT angiography (CTA) showed instead a very focal, severely stenotic SMA, 6 mm from its origin, with no evidence of atherosclerotic disease before or after, compatible with a very localized isolated dissection (**Fig. 2A**). There was no thrombosis in the arterial nor venous mesenteric system. Due to the persistence of symptoms, which prompted the vascular consult, the patient was consented and prepared for angiography and possible stenting.

**Figure 3:**
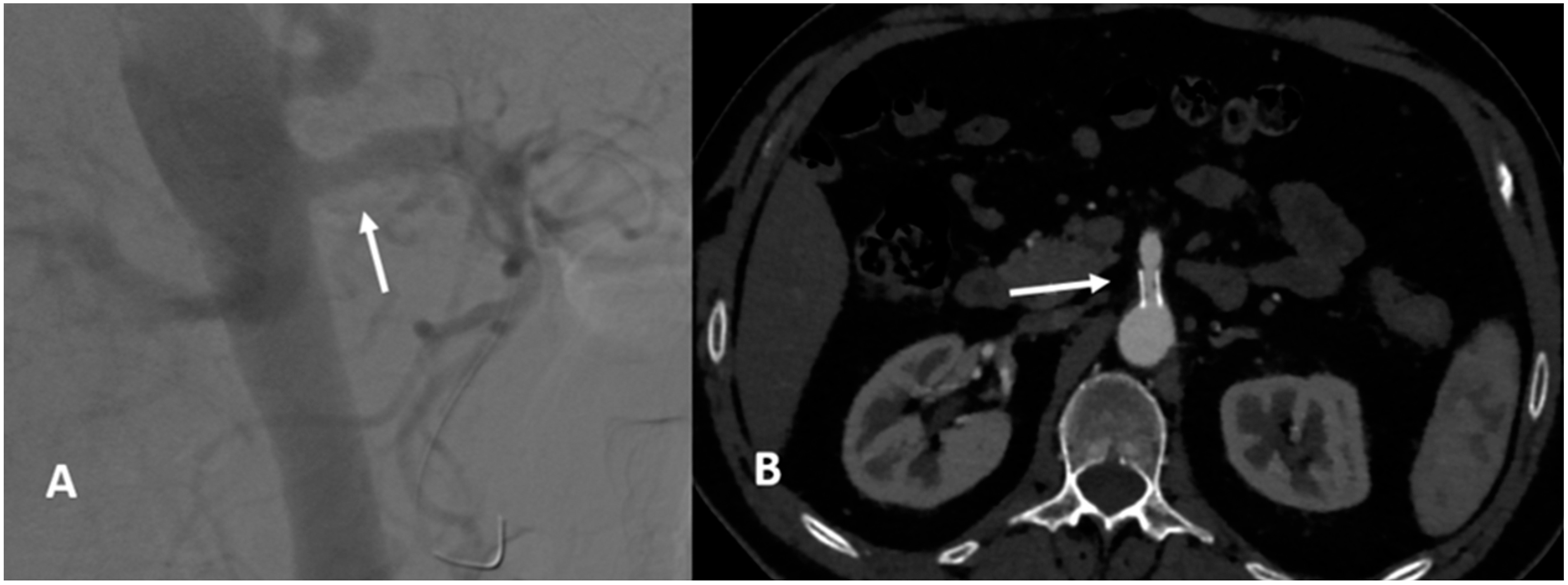
Fig. 3 (**A**) Angiography post-stenting showing patent superior mesenteric artery (SMA). (**B**) computed tomography angiography 6 months post repair. The SMA is patent, but there is evidence of minimal intimal hyperplasia.

Through a left brachial approach, aorto-mesenteric angiogram with anteroposterior and lateral views confirmed the presence of a very focal flap protruding into the SMA origin and compatible with dissection. The pig tail was then exchanged to a 6 French (Fr) Envoy guiding catheter (Cordis, Miami Lakes, FL, USA). Coaxial 4 Fr vertebral and 0.035 hydrophilic guide wires helped in crossing the narrowing; distal SMA angiography was done (**Figs. 2B** and **2C**). The system was exchanged to a 0.014 BMW wire (Abbott, Abbot Park, IL, USA), over which stenting was done using a 6.5×18-mm Herculink stent (Abbott, Abbot Park, IL, USA). Excellent angiographic results were obtained (**Fig. 3A**) with brisk, non-obstructed flow into the distal SMA. Heparin was administered during the procedure, keeping the activated clotting time at >250.

The patient was discharged home the next day on dual antiplatelet therapy for one year, followed by 75 mg clopidogrel. Symptoms resolved completely over the next few days post procedure.

**Figure 4:**
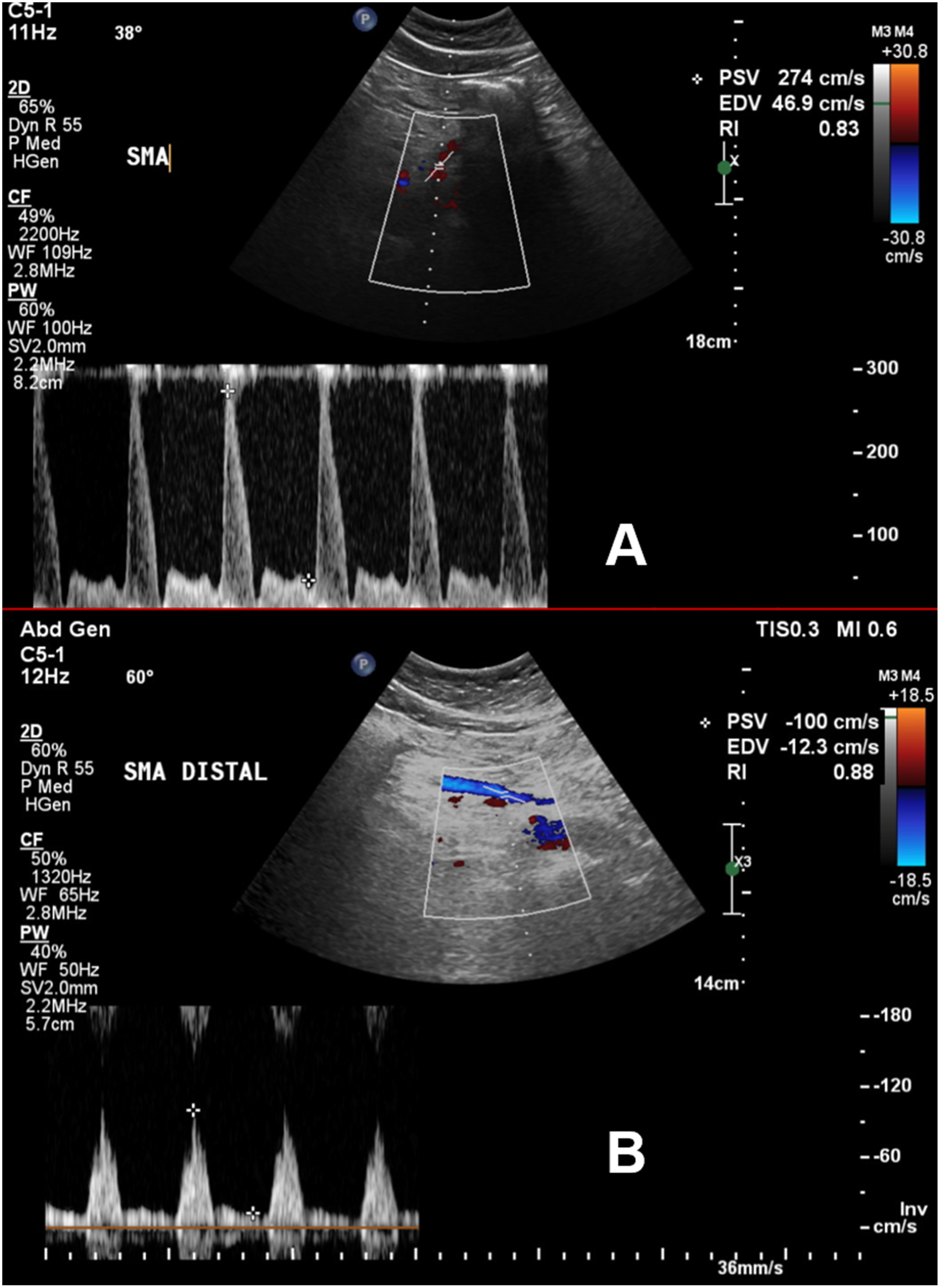
Fig. 4 (**A**) Duplex ultrasound 2 years post repair. There is increased velocity in the proximal superior mesenteric artery (SMA), indicating stent stenosis. (**B**) Duplex ultrasound 2 years post repair. The velocity is normal in the distal SMA, indicating patency and normal perfusion.

During the two years of follow-up, the patient had no complaints. A CT of the abdomen 6 months post-angioplasty showed patent stent with a thin line of intimal hyperplasia and reduction in the collateral network of the pancreaticoduodenal arcade (**Fig. 3B**). Since then, we followed-up the patient at 6-month intervals with arterial duplex scans. Although the stent remains patent, there is evidence of early, in-stent stenosis documented with increased velocities on the duplex scans. The distal SMA, however, continues to have normal flow and velocity waveform (**Fig. 4**), and the patient continues to be followed-up.

## Etiology

ISMAD is a rare arterial pathology that has been under investigation since 1947.^3)^ It is more common among males (88%) in their fifth decade of life.^6)^ Its etiology appears to be multifactorial, consisting of anatomic, genetic, and systemic components.

### Systemic

The concurrence of ISMAD with other isolated arterial dissections and aneurysms, such as celiac and renal arteries,^7–9)^ hints for a systemic component. Certain vascular diseases have already been linked to ISMAD, such as fibromuscular dysplasia, medial degeneration, and atherosclerosis.^10)^ There is also an increased risk of ISMAD with neoplasms, such as colon and gastric cancers,^11–13)^ which can be explained by the systemic neoplastic inflammation as well as the anatomic disturbance via mass effect.

### Anatomic

The SMA’s anatomy is an etiologic factor as well; certain variants increase its susceptibility to shear stress and therefore dissection. Shear stress develops as the SMA loses its mechanical support from the pancreas and bends freely within the mesenteric root.^14)^ The convexity of SMA after bending causes shear stress to push against the anterior wall, which happens to be the most common site of ISMAD.^15)^ Furthermore, it has been shown that as the angle between the aorta and the SMA’s convex curvature approaches 90°, shear stress and, by extension, ISMAD incidence, increases.^16,17)^ The involvement of SMA’s anatomy in ISMAD is supported by Kim’s computational simulation of blood flow. It illustrates accelerated hemodynamic flow at the transition site of SMA, which causes abnormal mechanical stress on the anterior wall and precipitate dissection.^18)^ It is noteworthy that in the case reported herein, the very first segment of the SMA is involved, whereby the ISMAD usually spares the first 1.5–3 cm.^15)^

### Genetic

The third causative agent in ISMAD is the genetic component, which has long been hypothesized because most cases reported have been from East Asia.^6)^ A recent case reported from China has strengthened this hypothesis, where a familial case of ISMAD was diagnosed and linked to a chromosomal locus of 5q13–14.^19)^

Our patient reported having neither personal nor family history that was relevant to the above predisposing factors, except for the blunt trauma sustained few days prior, before which he had no abdominal symptoms.

## Clinical Presentation

ISMAD’s clinical presentation is varied and elusive. Up to 33% of patients with ISMAD are asymptomatic and found incidentally.^9)^ Symptomatic patients often present with abdominal pain localized to the epigastrium, left hypochondrium, or umbilical region.^20,21)^ The abdominal pain can either be acute or chronically postprandial.^20,22)^ It can also be associated with nausea, vomiting, hematochezia, diarrhea, or back pain.^6)^ These nonspecific symptoms often delay the diagnosis of ISMAD, as in our patient, which puts patients at risk of several life-threatening complications. These include acute mesenteric ischemia, SMA aneurysm, hemorrhagic shock, peritonitis, and associated celiac artery compression syndrome.^6,23–25)^ Nevertheless, the mortality rate of ISMAD is at most 0.69%.^6)^

## Diagnosis

Diagnosis of ISMAD is as evasive as its clinical presentation. While angiography is the gold standard for diagnosing arterial pathologies, ultrasonography (US) and CT are used initially. US findings that suggest ISMAD are SMA dilation with luminal narrowing and presence of an intimal flap.^26)^ Although these findings can be missed,^22)^ a cohort study done by Bao et al. showed that color Doppler US is 95%sensitive, albeit the study included 19 patients only.^27)^ CT, on the other hand, shows an extended list of findings: SMA dilation, intimal flap, intramural hematoma, false lumen thrombosis, increased fat attenuation around the SMA, and mesenteric hematoma.^28)^ Furthermore, an added advantage of CT over US is the ability to classify ISMAD, which has the potential to drive the treatment of choice.

## Classification of ISMAD

There are five main classification systems used in ISMAD, all of which depend on imaging findings. The earliest classification system is Sakamoto’s, which classifies ISMAD into four: type I has a patent false lumen with re-entry site; type II has a patent false lumen without re-entry site; type III has a thrombosed false lumen with ulcer-like projection (ULP); and type IV has a thrombosed false lumen with no ULP.^29)^ Zerbib et al. modified Sakamoto’s classification and added type V, which has aneurysmal dissection and distal SMA stenosis, and type VIa and VIb, which have total and partial SMA thrombosis, respectively (**Tables 1** and **2**).^30)^

**Table 1:** Table 1 Sakamoto, Zerbib, and Li classifications of isolated superior mesenteric artery dissection

	Classification systems		
Types	Sakamoto	Zerbib	Li*
I	Patent false lumen with re-entry site		
II	Patent false lumen without re-entry site		
III	Thrombosed false lumen with ulcer-like projection		
IV	Thrombosed false lumen without ulcer-like projection		
V		Aneurysmal dissection, distal SMA stenosis	Dissecting aneurysm
VI a		Total SMA thrombosis	
VI b		Partial SMA thrombosis	

*Check **Table 2** for Li subtypes. SMA: superior mesenteric artery

**Table 2:** Table 2 Subtypes of types II, III, & IV in Li’s classification system of isolated superior mesenteric artery dissection

Subtype	
a	Patent true lumen
b	Stenosed true lumen
c	Occluded true lumen

A simpler classification system is Yun’s (**Table 3**). It categorizes ISMAD into three: false lumen with re-entry site (I); false lumen with no re-entry site that is either patent (IIa) or thrombosed (IIb); and occluded SMA (III).^31)^ However, Yun’s classification does not correlate with symptomatology; instead, it positively correlates with the dissection length.^31)^

**Table 3:** Table 3 Yun’s classification system of isolated superior mesenteric artery dissection

Yun’s Classification System	
I	False lumen with re-entry site
II a	Patent false lumen without re-entry site
II b	Thrombosed false lumen without re-entry site
III	Occluded SMA

SMA: superior mesenteric artery

Yun’s system can be combined with Luan’s, which classifies ISMAD based on its location rather than morphology (**Table 4**). Luan’s classification system categorizes ISMAD into four types: at the curved part and extending proximally (A), limited to the curved part (B), and at the curved part and extending distally (C), with possible involvement of the ileocolic or distal ileal artery (D).^32)^ Type B was the least symptomatic in Luan’s report whereas type D was the most.

**Table 4:** Table 4 Luan’s classification system of isolated superior mesenteric artery dissection

Luan’s Classification System	
A	Curved part of SMA extending proximally
B	Curved part of SMA without extending
C	Curved part of SMA extending distally
D	Involvement of ileocolic or distal ileal artery

SMA: superior mesenteric artery

The fifth classification system is Li’s (**Table 1**).^33)^ It adapts Sakamoto’s four types and subcategorizes types II, III, and IV into a, b, and c: patent true lumen, stenosed true lumen, and occluded true lumen, respectively (**Table 2**). It also adds type V (dissecting aneurysm), as in Zerbib’s modification. Hence, Li’s classification system is the most extensive and useful thus far, albeit it does not include all the findings found in the literature.

**Figure 5:**
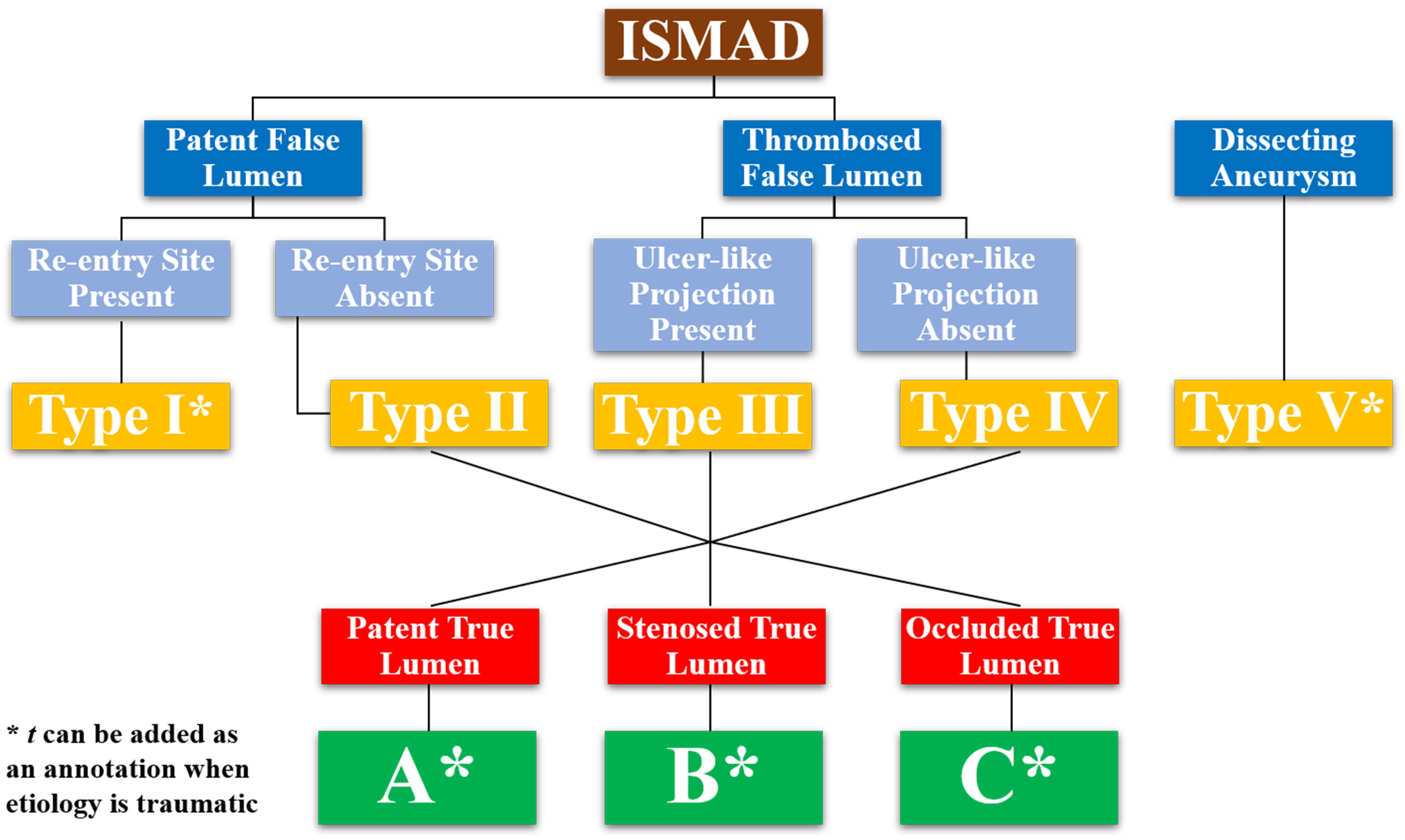
Fig. 5 Li’s classification system of isolated superior mesenteric artery dissection with a proposed addition of subclass *t* to indicate traumatic etiology.

In the case of our patient, the ISMAD had a thrombosed false lumen without an ULP and a stenosed true lumen. Therefore, according to Li’s classification, our index case would be categorized into type IVb. Yet the involvement of the SMA origin in blunt trauma tempts us to wonder whether a new subclass or category is required (**Fig. 5**).

## Treatment

The purpose of classifying ISMAD is to guide its management, which continues to be a controversial topic due to the lack of evidence-based guidelines. Surgical revascularization, despite being a definitive treatment, is often used as a last resort or in cases where intestinal ischemia is suspected.^34,35)^ Conservative management and endovascular approach are often used instead.

Conservative treatment is the first-line option in up to 87% of cases, with a success rate reaching up to 97%.^36,37)^ It primarily includes pain management, bowel rest, intravenous fluid administration, and hypertension control, with a length of stay reaching two weeks.^6,38)^ Antiplatelets and anticoagulants are occasionally added to the regimen as well,^39,40)^ although their therapeutic benefit is yet to be established. In their case series that included 25 patients, Liu et al. reported a beneficial effect of anticoagulants (70% vs. 17%),^41)^ whereas several studies with stronger evidence demonstrated their inefficacy.^31,36,42)^

Endovascular repair, such as stent placement and false lumen coiling,^37,43)^ is an available treatment for ISMAD. The candidacy for endovascular treatment is still unclear. Failure of conservative treatment, which can reach up to 16%, is the most common reason for escalating to endovascular repair.^44)^ Other advocated indications are pseudoaneurysms larger than 2 cm, aneurysmal changes, and significant stenosis of the true lumen.^33,45)^ Luminal stenosis, however, has been shown to respond to expectant management, and is therefore not adopted as an endovascular indication by most practitioners.^46–48)^ This comes in line with the latest published guidelines of the European Society for Vascular Surgery, which, because of the lack of high-quality unequivocal studies in support of early interventions, are still recommending conservative approach initially, with anticoagulation added for symptomatic patients and endovascular interventions for persistent or worsening symptoms.^49)^

Nevertheless, stenting has been shown recently to have a 99% patency rate and 95.8% event-free survival rate after five years, which is better than that of conservative management (event-free survival rate at three years is 62.5%),^50)^ in addition to an 88.3% complete remodeling of the vessel after stenting. Hence, are we watching a paradigm shift in the management in favor of routine invasive therapy?

Our index patient failed conservative treatment, including anticoagulation, since his pain persisted for more than two weeks. Thus, we decided to proceed with endovascular stenting, especially that the lesion was very focal; getting into the distal true lumen was simple, and no bridges were burnt, keeping the option of surgical intervention open in the future if need be.

Despite the high success rate, endovascular intervention carries its own complications. These include restenosis and early thrombosis, failure to stent an expanded dissection, deploying the stent across an aneurysmal false lumen, and occluding arteries that branch off the false lumen, which puts the intestines at risk of necrotic ischemia.^51–53)^

## Conclusion

ISMAD incidence is expected to increase with advancing imaging technology and utilization. Its symptoms are shared with other abdominal pathologies and should be considered in an acute or chronic setting, particularly with a history of blunt trauma. A dedicated CT of the abdomen is the diagnostic tool of choice, as it can rule out other etiologies and allow classification, to which a traumatic type may be added. Optimal management remains controversial given the lack of high-quality studies, but a conservative approach is applied initially, in which asymptomatic ISMAD is treated with antihypertensive and antiplatelet therapies, while anticoagulants are added for symptomatic cases. For patients with persistent symptoms or worsening aneurysm, an endovascular intervention is advised, keeping surgical revascularization as a last resort. Recently however, there has been a tendency to intervene earlier on symptomatic patients with excellent durable outcomes.
